# Integrated transcriptomics and proteomics assay identifies the role of FCGR1A in maintaining sperm fertilization capacity during semen cryopreservation in sheep

**DOI:** 10.3389/fcell.2023.1177774

**Published:** 2023-08-02

**Authors:** Jiachen Bai, Guizhen Zhou, Shaopeng Hao, Yucheng Liu, Yanhua Guo, Jingjing Wang, Hongtao Liu, Longfei Wang, Jun Li, Aiju Liu, Wendell Q. Sun, Pengcheng Wan, Xiangwei Fu

**Affiliations:** ^1^ Institute of Biothermal Science and Technology, School of Health Science and Engineering, University of Shanghai for Science and Technology, Shanghai, China; ^2^ National Engineering Laboratory for Animal Breeding, Beijing Key Laboratory for Animal Genetic Improvement, College of Animal Science and Technology, China Agricultural University, Beijing, China; ^3^ State Key Laboratory of Sheep Genetic Improvement and Healthy Breeding, Institute of Animal Husbandry and Veterinary Sciences, Xinjiang Academy of Agricultural and Reclamation Sciences, Shihezi, China; ^4^ Department of Animal Science, School of Life Sciences and Food Engineering, Hebei University of Engineering, Handan, China; ^5^ Department of Reproductive Medicine, Reproductive Medical Center, The First Hospital of Hebei Medical University, Shijiazhuang, China

**Keywords:** sperm, programmed freezing, multi-omics, FCGR1A, fertilization, sheep

## Abstract

Semen cryopreservation is a promising technology employed in preserving high-quality varieties in animal husbandry and is also widely applied in the human sperm bank. However, the compromised qualities, such as decreased sperm motility, damaged membrane structure, and reduced fertilization competency, have significantly hampered the efficient application of this technique. Therefore, it is imperative to depict various molecular changes found in cryopreserved sperm and identify the regulatory network in response to the cryopreservation stress. In this study, semen was collected from three Chinese Merino rams and divided into untreated (fresh semen, FS) and programmed freezing (programmed freezing semen, PS) groups. After measuring different quality parameters, the ultra-low RNA-seq and tandem mass tag-based (TMT) proteome were conducted in both the groups. The results indicated that the motility (82.63% ± 3.55% vs. 34.10% ± 2.90%, *p* < 0.05) and viability (89.46% ± 2.53% vs. 44.78% ± 2.29%, *p* < 0.05) of the sperm in the FS group were significantly higher compared to those in the PS group. In addition, 45 upregulated and 291 downregulated genes, as well as 30 upregulated and 48 downregulated proteins, were found in transcriptomics and proteomics data separately. Moreover, three integrated methods, namely, functional annotation and enrichment analysis, Pearson’s correlation analysis, and two-way orthogonal partial least squares (O2PLS) analysis, were used for further analysis. The results suggested that various differentially expressed genes and proteins (DEGs and DEPs) were mainly enriched in leishmaniasis and hematopoietic cell lineage, and Fc gamma receptor Ia (FCGR1A) was significantly downregulated in cryopreserved sperm both at mRNA and protein levels in comparison with the fresh counterpart. In addition, top five genes (*FCGR1A*, *HCK*, *SLX4*, *ITGA3*, and *BET1*) and 22 proteins could form a distinct network in which genes and proteins were significantly correlated (*p* < 0.05). Interestingly, FCGR1A also appeared in the top 25 correlation list based on O2PLS analysis. Hence, FCGR1A was selected as the most potential differentially expressed candidate for screening by the three integrated multi-omics analysis methods. In addition, Pearson’s correlation analysis indicated that the expression level of FCGR1A was positively correlated with sperm motility and viability. A subsequent experiment was conducted to identify the biological role of FCGR1A in sperm function. The results showed that both the sperm viability (fresh group: 87.65% ± 4.17% vs. 75.8% ± 1.15%, cryopreserved group: 48.15% ± 0.63% vs. 42.45% ± 2.61%, *p* < 0.05) and motility (fresh group: 83.27% ± 4.15% vs. 70.41% ± 1.07%, cryopreserved group: 45.31% ± 3.28% vs. 35.13% ± 2.82%, *p* < 0.05) were significantly reduced in fresh and frozen sperm when FCGR1A was blocked. Moreover, the cleavage rate of embryos fertilized by FCGR1A-blocked sperm was noted to be significantly lower in both fresh (95.28% ± 1.16% vs. 90.44% ± 1.56%, *p* < 0.05) and frozen groups (89.8% ± 1.50% vs. 82.53% ± 1.53%, *p* < 0.05). In conclusion, our results revealed that the downregulated membrane protein FCGR1A can potentially contribute to the reduced sperm fertility competency in the cryopreserved sheep sperm.

## 1 Introduction

Cryopreservation of semen has emerged as an important technology for efficient reproduction of livestock and poultry and protection of endangered species. It can aid to propagate animal offspring with excellent production performance, break the barriers to facilitate the exchange of genetic information caused by geographical isolation, and accelerate the promotion as well as application of *in vivo* and *in vitro* embryo production technologies (Kumar et al., 2019; Bolton et al., 2022). At present, slow freezing (also called programmed freezing) of semen has been widely used in animal breeding (Oldenhof et al., 2021) as well as in the donation and fertility preservation of human males (Tao et al., 2020; Agarwal et al., 2021).

It has been reported previously that cryoprotectant toxicity, osmotic pressure alteration, and ice crystal formation could inevitably induce distinct functional and structural damage in sperms during cryopreservation (Ezzati et al., 2020; Peris-Frau et al., 2020). Although optimized freezing procedures and the application of low-toxic cryoprotectants can help limit the formation of ice crystals and reduce the potential toxicity to the cell, decreased sperm viability as well as motility, reduced acrosome integrity, and compromised fertilization capability were observed due to the imbalance between the cooling rate and permeability velocity in the slow freezing program (Whaley et al., 2021). Moreover, it was reported that the elevated level of reactive oxygen species in the cryopreserved sperm could result in substantial DNA damage, plasma membrane damage, lipid peroxidation, and acrosomal membrane damage (Khan et al., 2021). Interestingly, a previous study has indicated that decreased DNA integrity induced by freezing could activate the repair pathway and alter the epigenetic reprogramming profile in the fertilized embryo (Wyck et al., 2018; Esteves et al., 2021). In addition, evidence has shown that both intact acrosomes and adequate sperm motility were required for proper fertilization (Yoshida et al., 2008). At the onset of the acrosome reaction, plasma membrane and acrosomal membrane were fused to form pores, which can release acrosome enzymes (Stival et al., 2016), and thus the intactness of the plasma/acrosomal membrane is pivotal for the successful acrosome reaction. Moreover, previous studies have also indicated that the plasma membrane intactness and acrosomal membrane integrity in the bovine cryopreserved sperm were reduced to 65.2% and 34.0%, respectively (Zoca et al., 2021). Furthermore, cryopreservation could also induce decreased sperm motility and viability (Estudillo et al., 2021). Interestingly, it was reported that decreased plasma membrane integrity could effectively lead to reduced sperm motility (Schulz et al., 2020). However, the molecular mechanisms underlying the compromised fertility in the cryopreserved sperm have not been fully identified yet.

Nowadays, significant progress has been made in understanding the sperm cryodamage mechanisms with the aid of high-throughput omics technologies. For instance, transcriptome analysis has revealed that numerous genes could be directly correlated with sperm fertility and freezing tolerance (Khan et al., 2021). Non-coding RNA sequencing of cryopreserved bull semen demonstrated that the differentially expressed miRNA and mRNA fragments were related to fertilization, ATP production, and apoptosis (Shangguan et al., 2020). miRNA sequencing of cryopreserved porcine semen indicated that various differentially expressed miRNAs were associated with energy metabolism, sperm structure, motility, and apoptosis (Zhang et al., 2017). The semen transcriptomes of porcine showed that DEGs, related to inflammation and apoptosis, spermatogenesis, autophagy, protein phosphorylation, and energy metabolism, were significantly upregulated in the semen with low freezability (Fraser et al., 2020). Furthermore, proteome determination of boar sperm and bull seminal plasma identifies cryogenic biomarkers to assess sperm motility (Moura and Memili, 2016; Parrilla et al., 2019). It has been established that compared to a single omics analysis, integrated multi-omics analysis is based on large and multidimensional data, which can substantially reduce bias as well as noise and identify the possible relationship between phenotypes and molecules (DNA, RNA, proteins, and metabolites) with greater precision (Krassowski et al., 2020). In fact, few prior studies have been performed on cryopreserved livestock sperm, especially sheep, using omics techniques. In recent years, integrated multi-omics analysis has emerged as the method of choice to decipher the cellular molecular information flow with spatial–temporal specificity (Subramanian et al., 2020). Thus, in order to comprehensively understand the regulatory network of RNA and protein in response to the cryopreservation stress, integrated ultra-low RNA-seq and TMT proteomics assay was applied in the present study. We expect that our result will definitely aid to provide novel ideas and theoretical basis for improving the cryopreservation technology of the sheep semen.

## 2 Materials and methods

### 2.1 Experimental design and semen sample collection

**FIGURE 1:**
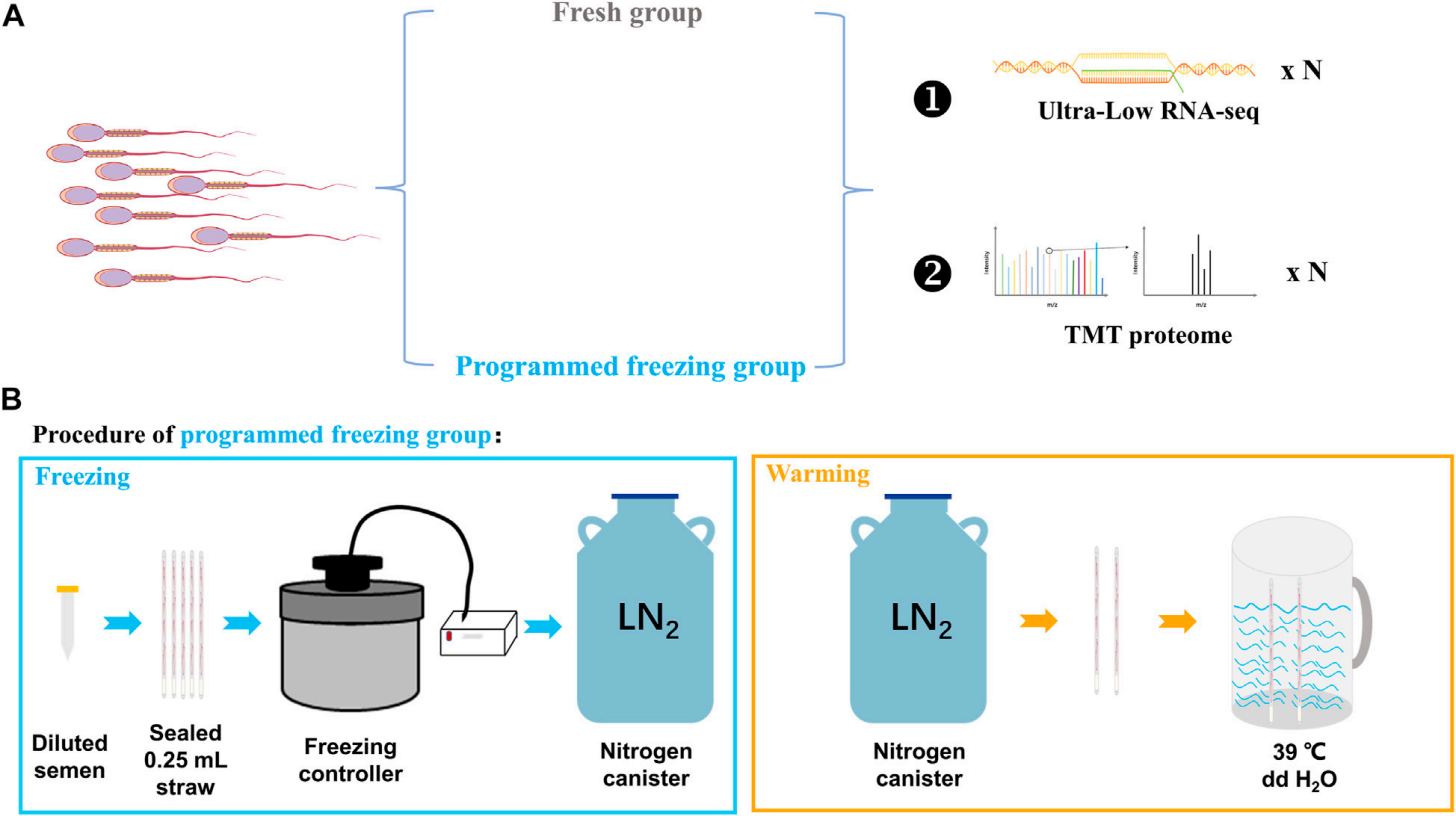
Experimental design for integrated multi-omics analysis before and after the programmed freezing semen in sheep. **(A)** Experimental grouping and omics analysis methods. **(B)** Schematic diagram depicting programmed freezing and thawing of sheep semen. ddH_2_O represents distillation–distillation H_2_O.

Three adult Chinese Merino rams with good nutrition, well-proportioned body, and normal libido were selected for the semen collection. All the rams were provided consistent feeding and management conditions with availability of free water. These rams were first trained in two semen collections, and finally the semen was collected for the third time. The collected semen was divided into two different parts: one was directly preprocessed before RNA and protein extraction (fresh semen, FS, *n* = 3), while the other was preprocessed after the freeze–thawing (programmed freezing semen, PS, *n* = 3). The RNA and proteins were extracted for ultra-low RNA-seq and TMT proteome determination (Figure 1A). However, only semen samples with motility higher than 80% in the FS group and higher than 30% in the PS group were used for omics analysis in the present study.

### 2.2 Semen-programmed cryopreservation and detection analysis

The collected fresh semen (sperm concentration: 2–3 × 10^9^ sperm/mL) was diluted at a ratio of 1:2 with the freezing Medium for Semen (IMV Technologies, Aigle, France). Furthermore, 200–220 μL diluted semen was pipetted from the centrifuge tube into 0.25-mL straws (IMV Technologies, Aigle, France) by using a modified 1-mL syringe. The open mouths of the straws were then sealed with tweezers heated using an alcohol lamp. After pre-cooling in a 4°C refrigerator freezer for 20 min, the sealed straws were thereafter inserted into slots of the freezer which have been pre-cooled with liquid nitrogen controlled using a freezing controller (CryoLogic CL5500TC, Australia) in advance. The freezing program of the freezing controller was that the temperature cooled down from 4°C to −30°C at a cooling rate of 3°C/min and from −30°C to −80°C at a cooling rate of 10°C/min (Supplementary Table S1). Finally, the straws were quickly removed from the freezing controller and stored in liquid nitrogen. For thawing, the sealed end of the straw was held with the forceps, removed from the liquid nitrogen, and shaken 2–3 times in the air. The straw was then quickly placed into a rewarming cup at 39°C for 30 s (Figure 1B). Finally, the rewarmed straw was incubated in a 38.5°C incubator for temporary storage.

The semen was diluted with the Freezing Medium for Semen to achieve the condition such that the sperm concentration was 3–5 × 10^7^ sperm/mL. A volume of 3 μL of semen was then pipetted onto an object slide from the upper-middle layers of the diluted semen. The sperm quality (viability, motility, velocity straight line, velocity curve line, average path velocity, and amplitude of lateral head) was measured for both FS and PS groups using the Mailang Animal Sperm Quality Analyzer (Nanning SongJing Tianlun Biotechnology Co., Ltd., Nanning, China).

### 2.3 RNA pretreatment, ultra-low RNA-seq, and DEG analysis

A volume of 400 μL of the diluted semen (sperm concentration: 6.6–10 × 10^8^ sperm/mL) was first centrifuged at 2,600 *g* for 3 min at room temperature, and then the supernatant was removed. A volume of 1 mL of physiological saline was added to resuspend the precipitate, and the mixture was centrifuged again at 2,600 *g* for 3 min, and then the supernatant was removed. The precipitate of the semen was mixed and resuspended in 500 μL of somatic cell lysis buffer (Allwegene, Beijing, China). After incubation for 5 min, the mixture was centrifuged at 10,000 *g* for 3 min; the supernatant was removed completely and stored in the liquid nitrogen.

The micro-amplification of RNA samples was carried out based on the instructions of the RNALib Single cell WTA Kit (Allwegene, Beijing, China). cDNA library construction was performed based on the instructions of Lifeint Transpose DNA Library Prep Kit for Illumina (Allwegene, Beijing, China). Thereafter, cDNAs, which could meet the quality requirements for the concentration and fragment size, were sequenced using the SE50 read length of the BGISEQ platform (Allwegene, Beijing, China). Fastp version 0.23.0, FastQC version 0.11.9, and MultiQC version 1.11 were used for cutting out the sequencing adapters and low-quality bases. HISAT version 2.2.1 was used to align various quality-controlled reads to the reference genome of *Ovis aries* (ARS-UI_Ramb_v2.0). After sorting using SAMtools version 1.12, the read numbers of the genes were counted using StringTie version 2.16. The DEGs were tested and compared using Student’s t-test and Benjamini and Hochberg (BH) method.

### 2.4 Protein pretreatment, TMT proteome determination, and quantitation of the proteins

A volume of 100 μL diluted semen (sperm concentration: 6.6–10 × 10^8^ sperm/mL) was initially directly mixed with 500 μL of 4°C pre-cooled normal saline and centrifuged at 100 *g* for 5 min, and then the supernatant was discarded. The precipitate of the semen was then mixed again with 300 μL of the pre-cooled normal saline and centrifuged at 1,000 *g* for 5 min, and thereafter the supernatant was discarded. The precipitate of the semen was mixed with 500 μL of 4% SDS solution in the centrifuge tube which was maintained at 95°C ddH_2_O for 10 min. After centrifugation at 13,800 *g* for 20 min, the supernatant was dispensed into cryovials and stored in liquid nitrogen.

Protein extraction and digestion, labeling, TMT labeling, and fractionation were performed by Allwegene Technology Inc., Beijing, China. LC-MS/MS analysis was carried out using a Q Exactive mass spectrometer coupled to the Easy-nLC system by Allwegene Company. The MS raw data for each sample were thereafter searched using Mascot version 2.2 (Matrix Science, London, United Kingdom) embedded into Proteome Discoverer 1.4 software to facilitate both identification and quantitation analysis. Different parameters and instructions of Mascot are summarized in Supplementary Table S2.

### 2.5 Separate analysis of RNA-seq and TMT proteome

PCoA and PCA were performed using the prcomp function of the R stats package. K-means clustering analysis of the samples of each omics was presented as heatmaps with dendrograms using the R package pheatmap. Kyoto Encyclopedia of Genes and Genomes (KEGG) and Gene Ontology (GO) enrichment analyses were performed using KOBAS (http://bioinfo.org/kobas/) for determining DEGs and DEPs. Gene set enrichment analysis (GSEA) was carried out on the identified clusters of the genes to reveal the functions of quantificational genes using OmicStudio tools (https://www.omicstudio.cn/tool). Clusters of Orthologous Groups of proteins and euKaryotic Orthologous Group (COG/KOG) classification analysis and subcellular localization analysis were performed at http://pantherdb.org/webservices/go/overrep.jsp.

### 2.6 Integrated multi-omics analysis between RNA-seq and TMT proteome

First, Co-KEGG analysis of DEGs and DEPs was carried out using KOBAS. Second, Pearson’s correlation analysis was used to analyze the correlation between different genes and proteins enriched for various significant pathways from single KEGG analysis of RNA-seq and TMT proteome. The |rho| > 0.8 and *p* < 0.05 were used as the potential thresholds for a significant correlation. Third, O2PLS analysis between RNA-seq and TMT proteome was performed at https://www.omicshare.com/tools/Home/Soft/o2pls. The top 25 joint loading genes and proteins were selected as the significant correlation components between the two omics. All the plotting was performed using OmicShare (https://www.omicshare.com/tools) and OmicStudio (https://www.omicstudio.cn/tool).

### 2.7 Blocking FCGR1A of sperm

The blocking was carried out based on a method described in a previous study (Rival et al., 2019) with some modifications. Briefly, 500 µL fresh semen (sperm concentration: 2–3 × 10^9^ spermatozoa per mL) was added to 500 µL of *in vitro* fertilization (IVF) solution and then centrifuged at 43 *g* for 3 min. The supernatant was transferred to 500 µL IVF solution and centrifuged at 160 *g* for 3 min, and thereafter the sediment was collected. For the frozen semen, 130 µL semen (sperm concentration: 6.6–10 × 10^8^ sperm/mL) was added to 1 mL of IVF solution after thawing and centrifuged at 43 *g* for 5 min, and thereafter the sediment was obtained. A volume of 100 μL of the sediment from fresh and frozen semen was used for the subsequent blocking experiments. For blocking, the anti-FCGR1A antibody (Bioss, Beijing, China) was incubated with semen (sperm concentration: 2–3 × 10^8^ sperm/mL) at a working concentration of 50 μg/mL for 30 min.

### 2.8 IVF and *in vitro* embryo culture

The sheep ovaries were collected from the local slaughterhouse. Cumulus–oocyte complexes (COCs) were obtained via aspiration of the follicles with 3–6 mm size. Thereafter, the COCs were subjected to *in vitro* maturation for 24 h at 38.5°C and 5% CO_2_. A volume of 3 μL semen (sperm concentration: 2–3 × 10^8^ sperm/mL) was added to 80 μL droplet containing 35–40 oocytes for fertilization. After 18 h of IVF, the embryos were finally washed and transferred to the IVC solution to facilitate embryonic development.

### 2.9 Statistical analysis

Statistically significant differences for various sperm quality parameters, cleavage rates, and blastocyst rates were determined using Student’s t-test. All statistical analyses were carried out using SPSS version 24.0 (IBM Corporation, Armonk, USA), and the data are presented as mean ± SEMs. *p* values <0.05 were considered significant, and *p* values <0.001 were considered extremely significant.

## 3 Results

### 3.1 Sperm quality parameters before and after programmed freezing of the semen

The results indicated that sperm motility (82.63% vs. 34.10%, *p* < 0.001) and viability (89.46% vs. 44.78%, *p* < 0.001) of the FS group were significantly higher than those of the PS group. The sperm motion parameters including velocity curve line (87.71 um/s vs. 68.39 um/s, *p* < 0.05), average path velocity (61.99 um/s vs. 48.36 um/s, *p* < 0.05), and amplitude of lateral head (25.69 um vs. 20.03 um, *p* < 0.05) were also significantly higher in the FS group compared to those of the PS group. However, the velocity straight line (43.65 um/s vs. 38.42 um/s, *p* > 0.05) was not significantly different between the two groups (Table 1).

**TABLE 1:** Sperm quality parameters before and after the programmed freezing semen.

Parameter	FS group	PS group
Sperm motility (%)	82.63 ± 3.55^A^	34.10 ± 2.90^B^
Sperm viability (%)	89.46 ± 2.53^A^	44.78 ± 2.29^B^
Velocity straight line (VSL) (um/s)	43.65 ± 3.43^a^	38.42 ± 2.40^a^
Velocity curve line (VCL) (um/s)	87.71 ± 6.85^a^	68.39 ± 5.24^b^
Average path velocity (VAP) (um/s)	61.99 ± 4.85^a^	48.36 ± 3.70^b^
Amplitude of the lateral head (ALH) (um)	25.69 ± 2.01^a^	20.03 ± 1.54^b^

^a^ Different capital letters on the same row indicate a highly significant difference (*p* < 0.001), whereas different lowercase letters on the same row indicate a significant difference (*p* < 0.05). The same letters on the same row indicate a non-significant difference (*p* > 0.05).

### 3.2 Overview of RNA-seq data and analysis of DEGs

**FIGURE 2:**
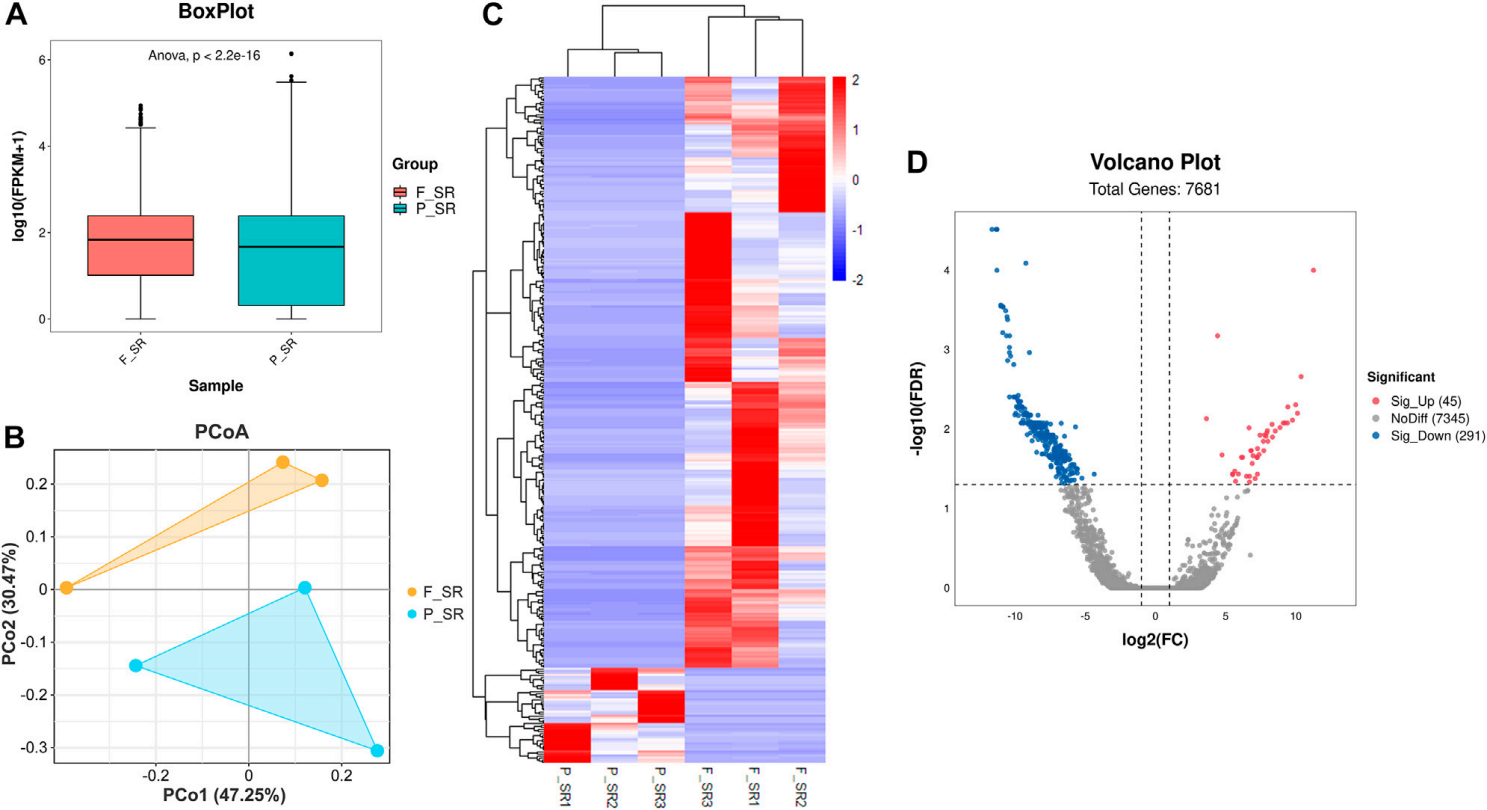
Quality assessment of RNA-seq data and the subsequent analysis of DEGs. **(A)** Boxplot of RNA-seq differences between FS and PS groups. **(B)** PCoA distribution of the samples obtained from FS and PS groups. **(C)** Heatmap of expression comparison of DEGs and clustering of the samples obtained from FS and PS groups. **(D)** Volcano plot of various quantificational genes for RNA-seq compared with FS and PS groups. F_SR represents the sample from RNA-seq of the FS group. P_SR represents the sample from RNA-seq of the PS group. Sig_Up represents the upregulated DEGs. Sig_Down represents the downregulated DEGs.

After the quality control of RNA-seq data, total reads of RNA-seq were aligned and counted. Among the total 310,699,628 reads, 186,669,343 (60.08%) unique mapped reads were obtained (Supplementary Table S3). Variance analysis of fragments per kilobase of the exon model per million mapped fragments (FPKM) for all the genes indicated that a significant difference could be found between FS and PS groups (*p* < 0.001) (Figure 2A). The six samples of the two groups were distributed in two different triangular areas under PCo1 (47.25%) and PCo2 (30.47%) after projection of the multidimensional gene expression data (Figure 2B). K-means clustering analysis indicated that the six samples were correctly clustered in both FS and PS groups through the centralization and normalization of the read numbers of DEGs (Figure 2C). A total of 7,682 genes were then identified from RNA-seq. Additionally, under the threshold value of Log_2_|fold change| > 1 and false discovery rate (FDR) < 0.05, 45 upregulated and 291 downregulated genes were obtained (Figure 2D). The top 20 DEGs are listed in Table 2.

**TABLE 2:** Top 20 DEGs obtained from RNA-seq data.

Gene name	Description	Log_2_ (fold change)	FDR (*p* adjusted)
CARNMT1	Carnosine N-methyltransferase 1	−11.28202062	3.07E-05
LPCAT4	Lysophosphatidylcholine acyltransferase 4	−11.64424531	3.07E-05
PLS1	Plastin 1	−11.3159436	3.07E-05
THAP4	THAP domain containing 4	−11.33720252	3.07E-05
RPSA	Small subunit ribosomal protein SAe	−9.229326988	8.19E-05
RBM44	RNA-binding motif protein 44	11.24917887	0.0001005
TMEM245	Transmembrane protein 245	−11.29318644	0.0001005
GEN1	GEN1 Holliday junction 5′-flap endonuclease	−11.01417458	0.000275
PLK2	Polo-like kinase 2	−11.00921955	0.0002859
LOC106990489	60 S ribosomal protein L21-like	−10.87339307	0.0002874
NECAP2	NECAP endocytosis associated 2	−10.84681499	0.0002911
SLX4	Structure-specific endonuclease subunit	−10.66750406	0.0003253
LOC105616164	-	−10.57128419	0.0003871
LOC101112729	-	−10.53273872	0.0004203
TEKT2	Tektin-2	−10.87612847	0.0006137
CDYL2	Chromodomain protein, Y-like 2	−10.61038298	0.0006707
LOC114115287	-	4.425679274	0.0006707
LONRF1	LON peptidase N-terminal domain and ring finger 1	−10.3890279	0.0006707
LYSMD2	LysM, putative peptidoglycan-binding domain containing 2	−10.39857244	0.0009386
ANP32B	Acidic leucine-rich nuclear phosphoprotein 32 family member B	−10.40244919	0.001089836

^a^ Gene name is the official gene symbol on NCBI (https://www.ncbi.nlm.nih.gov/). 3.07E-05 represents 3.07 × 10^−5^. 8.19E-05 represents 8.19 × 10^−5^.

### 3.3 Functional enrichment analysis

**FIGURE 3:**
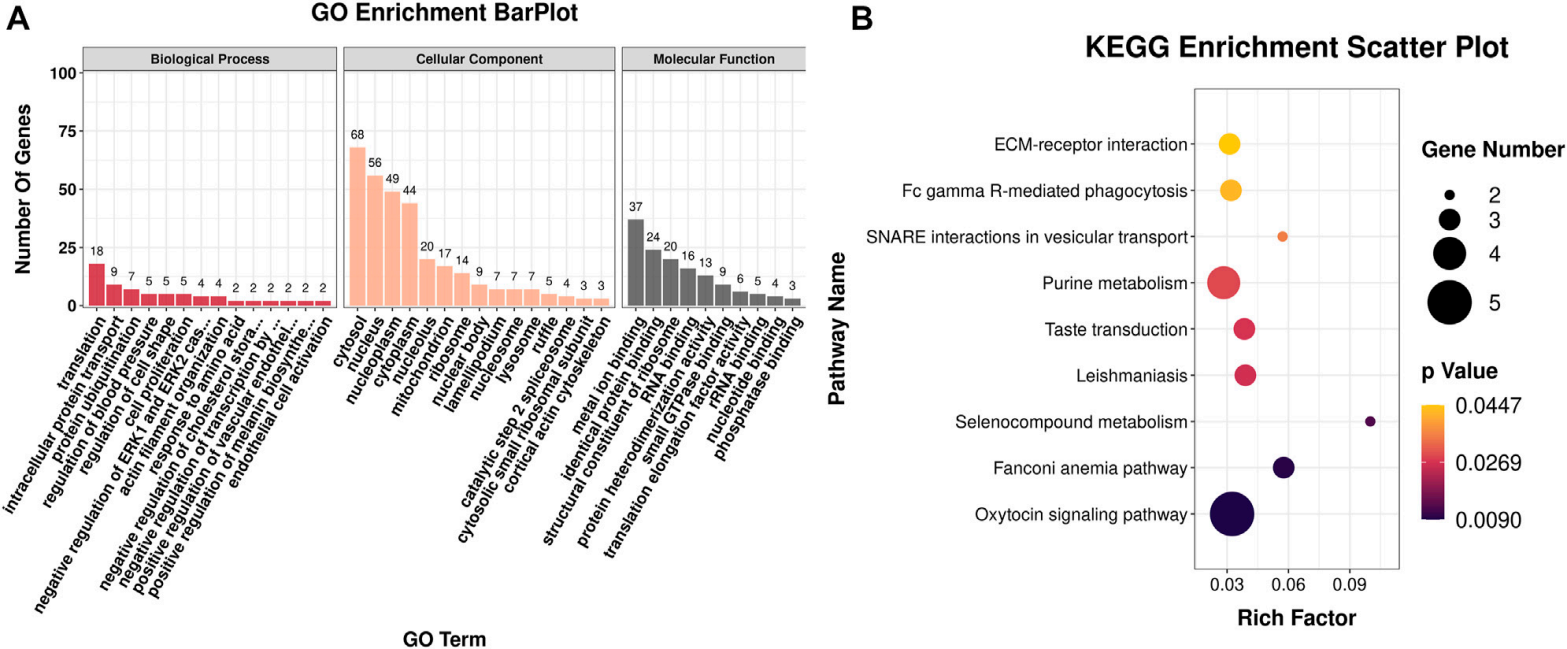
GO classification and KEGG enrichment analyses of DEGs obtained from RNA-seq. **(A)** Bar plot of GO classification of DEGs. **(B)** Scatter plot of the KEGG enrichment pathways of DEGs.

**FIGURE 4:**
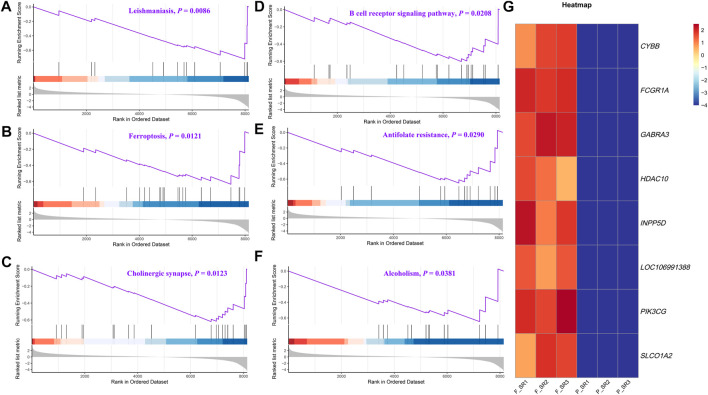
GSEA of the quantificational genes from RNA-seq. Representative GSEA significance pathways of gene sets, including those of leishmaniasis **(A)**, ferroptosis **(B)**, cholinergic synapse **(C)**, B-cell receptor signaling pathway **(D)**, antifolate resistance **(E)**, and alcoholism **(F)**. **(G)** Heatmap of the relative expression of DEGs enriched in significant pathways by GSEA.

The DEGs were then subjected to GO classification and KEGG enrichment analyses. GO classification analysis revealed that the number of DEGs distributed in the translation biological process (BP), intracellular protein transport BP, cytosol cellular component (CC), nucleus CC, metal ion-binding molecular function (MF), and identical protein-binding MF was 18, 9, 68, 56, 37, and 24, respectively (Figure 3A; Supplementary Table S4). A total of nine different significant pathways were identified from the KEGG enrichment analysis including oxytocin signaling pathway, Fanconi anemia pathway, and selenocompound metabolism. (Figure 3B; Supplementary Table S5). GSEA based on the quantificational genes indicated that the differential gene sets were mainly enriched in leishmaniasis, ferroptosis, cholinergic synapse, B-cell receptor signaling pathway, antifolate resistance, and alcoholism (Figures 4A–F; Supplementary Table S6). In addition, various DEGs, including *CYBB*, *FCGR1A*, *GABRA3*, *HDAC10*, *INPP5D*, *LOC106991388*, *PIK3CG*, and *SLCO1A2*, were also involved in these pathways (Figure 4G).

### 3.4 Assessment of TMT proteome data quality

**FIGURE 5:**
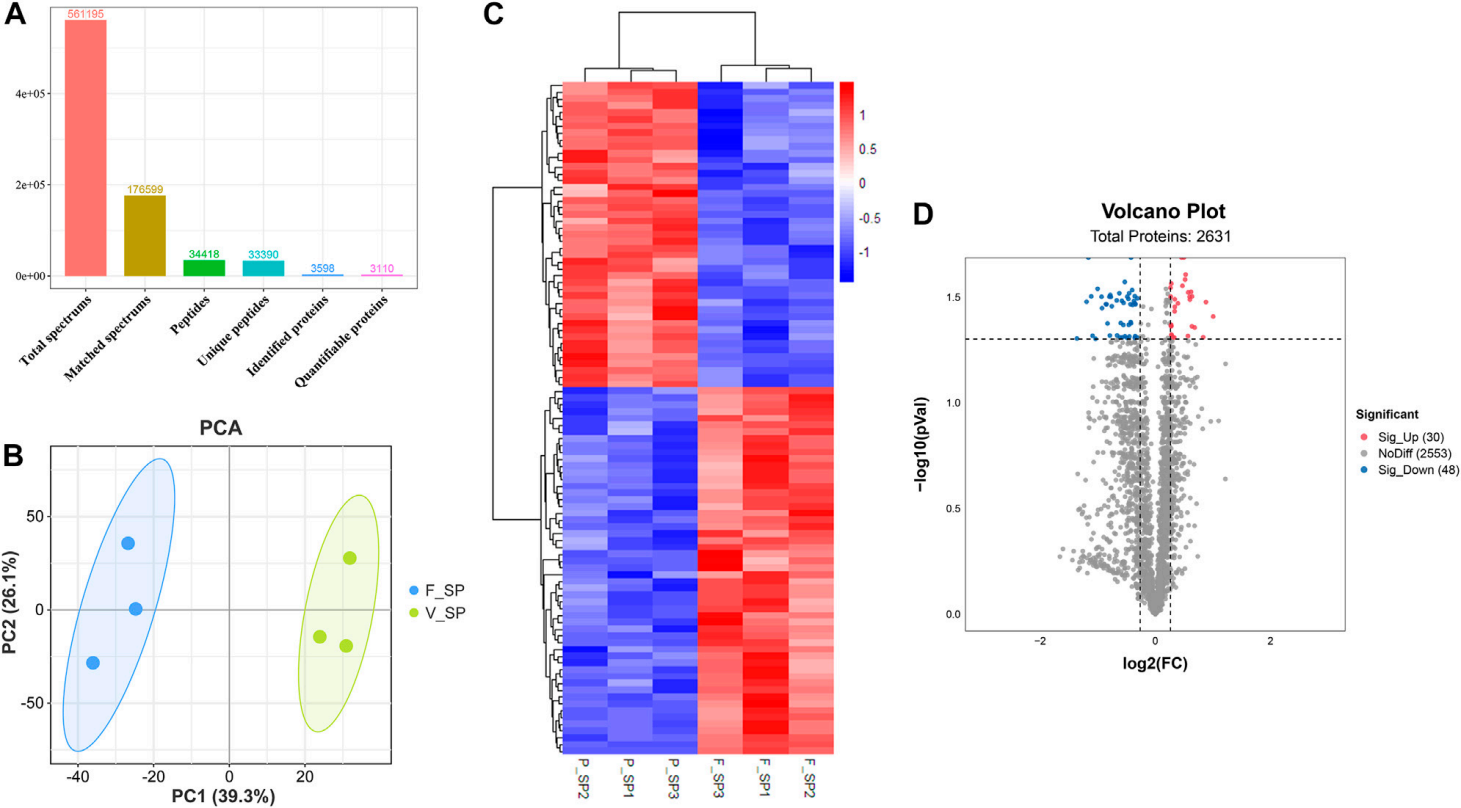
Quality assessment of TMT proteome data and analysis of DEPs. **(A)** Key information for each procedure of the proteome analysis. **(B)** PCA distribution of the samples from FS and PS groups. **(C)** Expression comparison of proteins and clustering of the samples from FS and PS groups. **(D)** Volcano plot of various quantificational proteins for proteome obtained from FS and PS groups. F_SP represents the sample from TMT proteome of the FS group. P_SP represents the sample from TMT proteome of the PS group. Sig_Up represents the upregulated DEPs. Sig_Down represents the downregulated DEPs.

After quality control, a total of 926,536 spectrums were acquired from TMT proteome, including 11,357 unique peptides and 2,639 identified proteins. Finally, 2,634 quantifiable proteins were obtained (Figure 5A). PCA demonstrated that six samples from FS and PS groups were aggregated within the group and then separated between the groups under PC1 (39.3%) and PC2 (26.1%) after projection of the multidimensional protein expression data (Figure 5B). K-means clustering analysis of DEPs also confirmed the PCA results (Figure 5C). A total of 2,631 proteins were identified from TMT proteome. Under the threshold value of Log_2_|fold change|> 0.263 and FDR <0.05, 30 up- and 48 downregulated proteins were obtained (Figure 5D). The top 20 DEPs are listed in Table 3.

**TABLE 3:** Top 20 DEPs derived from TMT proteome.

UniProt ID	Protein name	Description	Log_2_ (fold change)	FDR (*p* adjusted)
W5P906	DPP4	Dipeptidyl peptidase 4	−1.162410726	0
A0A6P7D504	PPP2R5C	Serine/threonine protein phosphatase 2a	−0.421287159	0
A0A6P7E8M7	LOC114115593	Testis development-related protein-like	0.45548898	0
A0A835ZQ00	-	U3 small nucleolar RNA-interacting protein 2	0.494694352	0
A0A836A4E5	-	Mitochondrial import receptor subunit TOM34	0.526037653	0.0248
A0A836AIY4	-	Proline-rich protein 30	0.516557038	0.02616667
A0A6P3E8Q6	PARK7	Parkinson protein 7	−0.530060172	0.02685714
A0A6P3TCD1	FAAH	Fatty-acid amide hydrolase	0.276976744	0.02730435
A0A836AK13	-	SPATA31 subfamily D member 1	0.470494131	0.028
A0A836D3F5	-	Tctex1 domain-containing protein 2	0.263514916	0.02854545
W5PUL3	UCHL1	Ubiquitin carboxyl-terminal hydrolase	−1.001612941	0.02892308
A0A6P7E699	SGTA	-	−0.402269542	0.02933333
W5Q603	SPACDR	Chromosome 24 C7orf61 homolog	0.620095909	0.02990476
W5PSY7	-	BRCA-2_OB1 domain-containing protein	0.57042112	0.03008
A0A6P3TGM3	GALT	Galactose-1-phosphate uridylyltransferase	−0.62445267	0.03033962
A0A836AFX3	-	Disintegrin and metalloproteinase domain-containing protein 20-like	−0.453971366	0.03034483
W5QHL1	FCGR1A	Fc fragment of IgG	−0.781876862	0.03092308
A0A6P3TG31	PSMA8	Proteasome subunit alpha-type 8	−0.356472135	0.03133333
A0A836A330	-	Brain acid-soluble protein 1	−1.112150699	0.0314
A0A836D6I1	-	Protein FAM75A2	0.636788587	0.031428571

^a^ Information about the proteins can be retrieved on UniProt (https://www.uniprot.org/) using UniProt ID.

### 3.5 Functional enrichment analysis of DEPs

**FIGURE 6:**
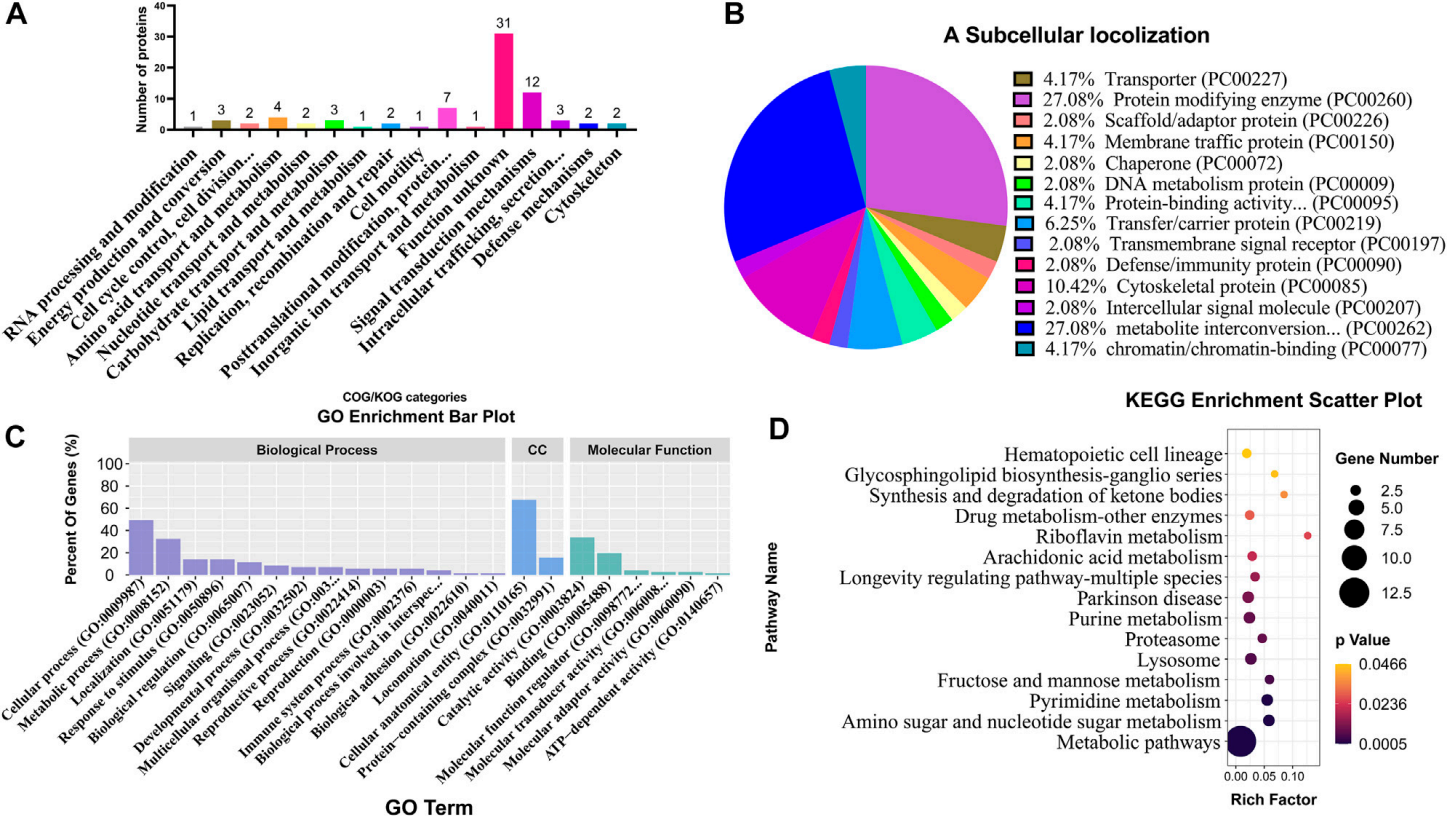
Classification and enrichment analyses of different DEPs from TMT proteome. COG/KOG classification analysis **(A)**, subcellular localization analysis **(B)**, GO classification analysis **(C)**, and KEGG enrichment analysis **(D)** of DEPs. CC represents cellular components.

COG/KOG category analysis indicated that 12 and 7 DEPs were mainly involved in signal transduction mechanisms and post-translational modification, respectively (Figure 6A; Supplementary Table S7). The subcellular localization analysis suggested that DEPs mainly belonged to the protein-modifying enzyme (27.08%) and metabolite interconversion enzyme (27.08%) (Figure 6B; Supplementary Table S8). Based on BP, CC, and MF classifications, proteins were mainly found to be involved in the cellular process BP, metabolic process BP, cellular anatomical entity CC, catalytic activity MF, and bind MF with the percentage of 49.3%, 32.4%, 67.6%, 33.8% and 19.7%, respectively (Figure 6C; Supplementary Table S9). KEGG enrichment analysis revealed that various DEPs were enriched in different metabolic pathways, such as amino sugar and nucleotide sugar metabolism, pyrimidine metabolism, fructose and mannose metabolism, and lysosome (Figure 6D; Supplementary Table S10).

### 3.6 Functional enrichment analysis between DEGs and DEPs

**FIGURE 7:**
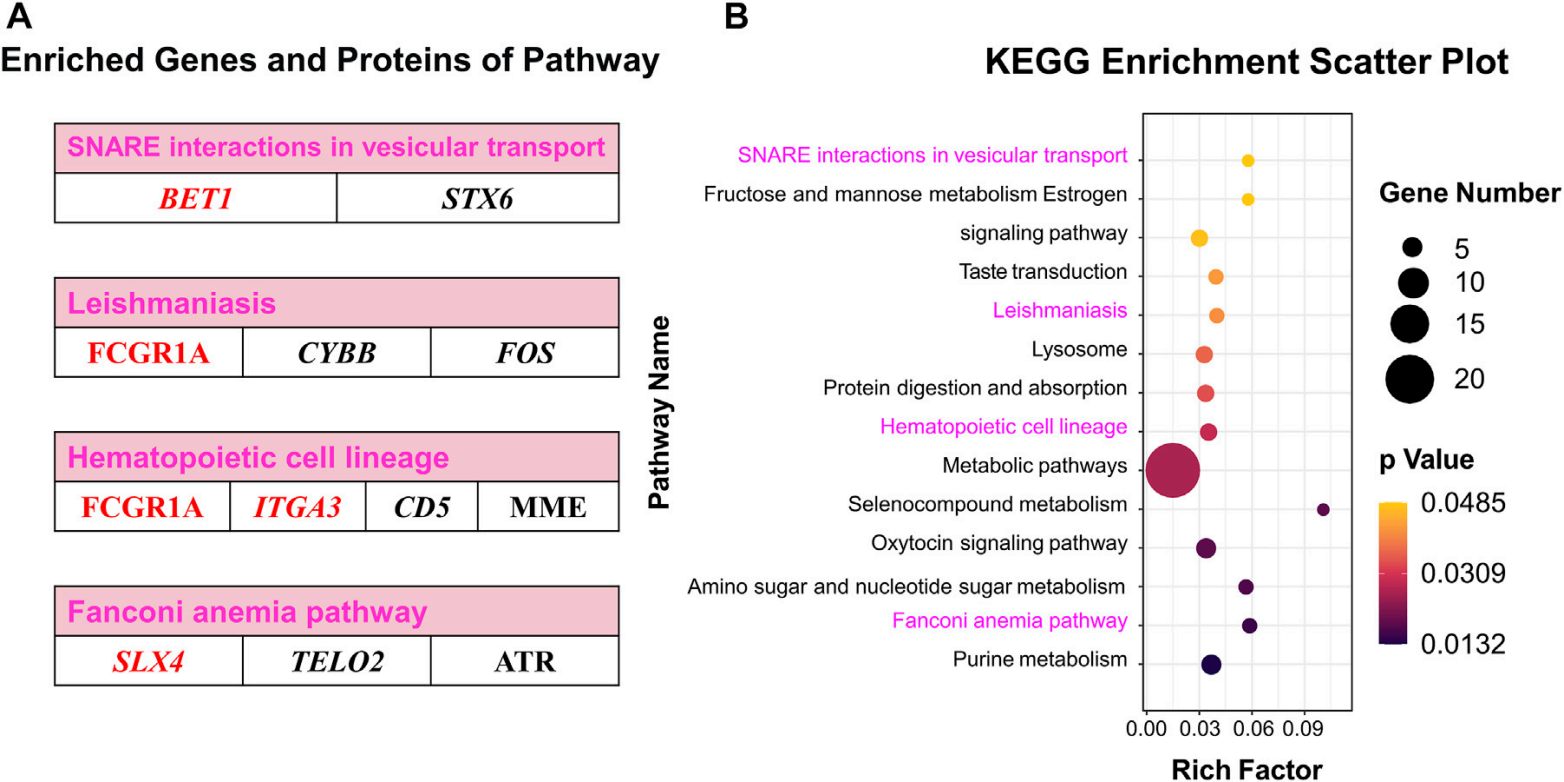
Co-KEGG enrichment analysis and specific DEGs and DEPs contained in significant pathways. **(A)** Enriched genes and proteins in interesting pathway. **(B)** Scatter plot of the Co-KEGG enrichment pathways of DEGs and DEPs. Words in red represent interesting genes or proteins.

Co-KEGG enrichment analysis of DEGs and DEPs revealed that the differentially expressed candidates were involved in 14 different pathways (*p* < 0.05; Figure 7A). Among these 14 pathways, four major pathways were enriched: SNARE interactions involved in the vesicular transport (*BET1* and *STX6*), leishmaniasis (*FCGR1A*, *CYBB*, and *FOS*), hematopoietic cell lineages (*FCGR1A*, *ITGA3*, *MME*, and CD5) and Fanconi anemia pathway (*SLX4*, *ATR*, and *TELO2*) (Figure 7B; Supplementary Table S11).

### 3.7 Pearson’s correlation analysis of the pathway-enriched DEGs and DEPs

**FIGURE 8:**
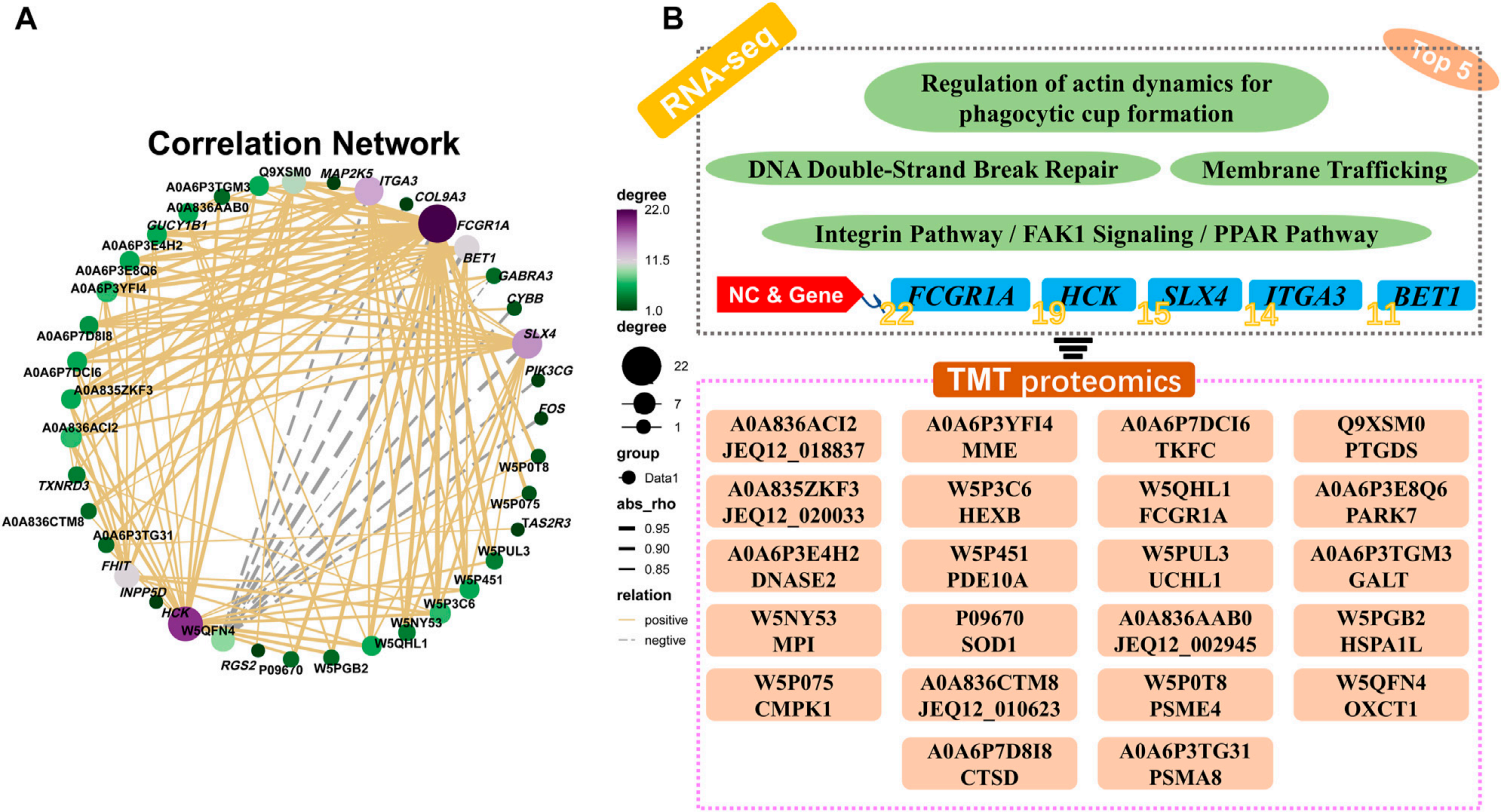
Pearson’s correlation analysis and the relational network between pathway-enriched DEGs and DEPs. **(A)** Pearson’s correlation analysis of DEGs and DEPs in significant pathways obtained by KEGG enrichment analyses of RNA-seq and TMT proteome. **(B)** Potential relationships and functional pathways of DEGs and DEPs.

Pearson’s correlation analysis indicated that there were 22 distinct proteins (JEQ12_018837, MME, TKFC, PTGDS, JEQ12_020033, HEXB, FCGR1A, PARK7, DNASE2, PDE10A, UCHL1, GALT, MPI, SOD1, JEQ12_002945, HSPA1L, CMPK1, JEQ12_010623, PSME4, OXCT1, CTSD, and PSMA8) which were significantly correlated with the top five genes: *FCGR1A*, *HCK*, *SLX4*, *ITGA3*, and *BET1*. The number of proteins significantly associated with *FCGR1A*, *HCK*, *SLX4*, *ITGA3*, and *BET1* was 22, 19, 15, 14, and 11, respectively (Figures 8A,B; Supplementary Table S12).

### 3.8 O2PLS of all the quantificational genes and proteins from RNA-seq and TMT proteome

**FIGURE 9:**
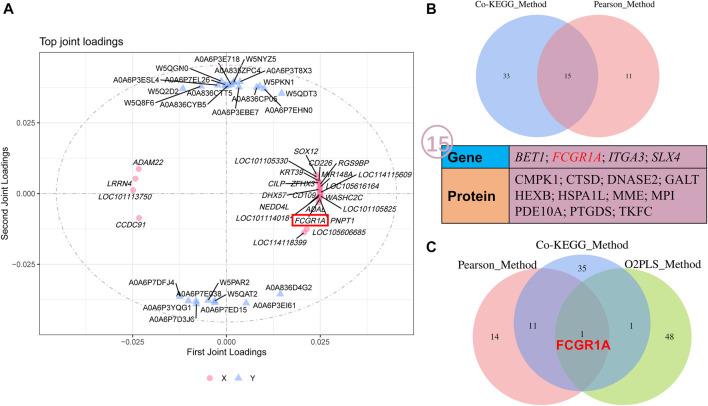
O2PLS analyses of all the quantificational genes and proteins derived from RNA-seq and TMT proteome. **(A)** O2PLS analysis of all the quantificational genes and proteins. **(B)** Collection of various genes and proteins between Co-KEGG enrichment analysis and Pearson’s correlation analysis. **(C)** Collection of genes and proteins between Co-KEGG enrichment analysis, Pearson’s correlation analysis, and O2PLS analysis. KEGG_Method represents Co-KEGG enrichment analysis. Pearson_Method represents Pearson’s correlation analysis. O2PLS_Method represents O2PLS analysis.

O2PLS analysis was performed between all the quantificational genes and proteins obtained from RNA-seq and proteome analyses. Thereafter, the top 25 genes and proteins contributing to the changes after cryopreservation were plotted (Figure 9A; Supplementary Table S13). Interestingly, FCGR1A appeared in both the top 25 gene and protein lists. Notably, FCGR1A was included in the list of 15 DEGs and DEPs (BET1, FCGR1A, ITGA3, SLX4, CMPK1, CTSD, DNASE2, GALT, HEXB, HSPA1L, MME, MPI, PDE10A, PTGDS, and TKFC) based on Co-KEGG enrichment analysis and Pearson’s correlation analysis (Figure 9B). In addition, it was also the only gene screened by three integrated multi-omics analysis methods (Figure 9C).

### 3.9 Correlation of FCGR1A with fertilization ability

**FIGURE 10:**
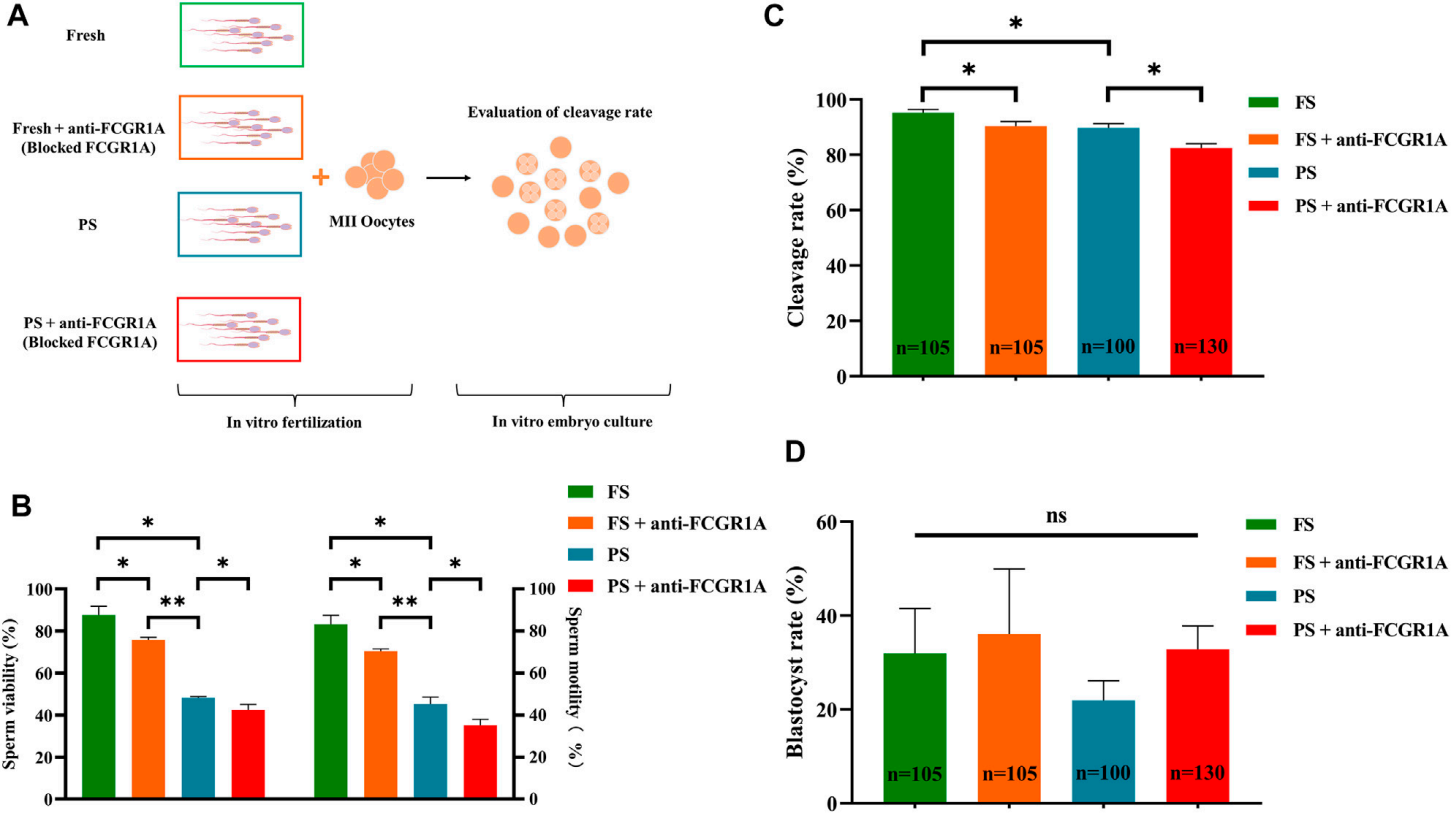
Correlation of FCGR1A with sperm fertilization ability. **(A)** Experimental design for the evaluation of the fertilization ability of FCGR1A-blocked sperm. **(B)** Difference between the sperm motility and viability after IVF with sperm obtained from different treatments. **(C)** Difference between the cleavage rate after IVF with sperm obtained from the different treatments. **(D)** Difference between blastocyst rates after *in vitro* fertilization with sperm from different treatments. FS represents fresh sperm after floating. FS + anti-FCGR1A represents fresh sperm blocked by anti-FCGR1A. PS represents programmed freezing sperm after floating. PS + anti-FCGR1A represents fresh sperm blocked by anti-FCGR1A. *p < 0.05, **p < 0.001; ns, non-significance.

We first evaluated the viability and motility after sperm preparation, and the results indicated that the sperm viability (87.65% ± 4.17% vs. 48.15% ± 0.63%, *p* < 0.001) and motility (83.27% ± 4.15% vs. 45.31% ± 3.28%, *p* < 0.001) of the fresh group was significantly higher than those of the frozen counterpart (Figures 10A,B). Pearson’s correlation analysis showed that both the sperm motility and viability were positively correlated with FCGR1A abundance based on omics data (Table 4; *p* < 0.05). Furthermore, blocking experiments were performed, and the result suggested that the viability (87.65% ± 4.17% vs. 75.8% ± 1.15%, *p* < 0.05) as well as motility (83.27% ± 4.15% vs. 70.41% ± 1.07%, *p* < 0.05) was significantly reduced in the fresh sperm blocked with the FCGR1A antibody. Interestingly, the dramatically decreased viability (48.15% ± 0.63% vs. 42.45% ± 2.61%, *p* < 0.05) and motility (45.31% ± 3.28% vs. 35.13% ± 2.82%, *p* < 0.05) were also observed in the frozen sperm blockage (Figures 10A,B). Moreover, when FCGR1A was blocked, the cleavage rate of embryos derived from either the fresh sperm (95.28% ± 1.16% vs. 90.44% ± 1.56%, *p* < 0.05) or the frozen sperm (89.8% ± 1.50% vs. 82.53% ± 1.53%, *p* < 0.05) was significantly lower compared to that of the corresponding unblocked counterparts (Figure 10C). However, no significant difference in the blastocyst formation rate was observed (Figure 10D).

**TABLE 4:** List of possible correlations between the sperm motility, viability, and FCGR1A expression level.

	*FCGR1A* (rho)	FCGR1A (rho)
Sperm viability	0.9859 (*p* = 0.0003)	0.9738 (*p* = 0.0010)
Sperm motility	0.9822 (*p* = 0.0005)	0.9661 (*p* = 0.0017)

^a^ Rho represents Pearson’s correlation coefficient.

## 4 Discussion

During programmed freezing, the stresses originating from multiple sources could lead to substantial structural damage and metabolic changes in sperm, which subsequently might impair sperm motility and fertilization ability. However, the molecular mechanisms underlying compromised fertilization competency have not been identified yet. In the present study, we have identified that FCGR1A was primarily responsible for the decreased sperm fertilization capacity using integrated transcriptomics and proteomics assays.

It has been reported previously that compared to the fresh semen, the total motility as well as progressive motility of the semen was significantly reduced after programmed freezing in Slovak dairy, Native Wallachian, and Improved Wallachian sheep rams (Vozaf et al., 2022). In consistent with the previous studies, we also discovered that severe cryodamage could effectively directly lead to reduced viability and motility in sheep. A number of prior studies have also indicated that the plasma and acrosome membranes can play an essential role in the capacitation, acrosome reaction, and sperm–oocyte recognition (Ritagliati et al., 2018; Bianchi and Wright, 2020). In addition, sperm motility is associated with ion channel proteins at the plasma membrane that can regulate potential and pH of the plasma membrane, which are essential for sperm fertilization (Nowicka-Bauer and Szymczak-Cendlak, 2021). However, till date, the specific mechanisms underlying sperm fertilization ability remain unclear.

To thoroughly illustrate the regulatory network involved in reduced sperm fertilization due to cryopreservation stress, integrated transcriptome and proteome analysis was first performed in the current study. A large number of up- and downregulated DEGs and DEPs were identified. It has been established that mature sperm are often in a state of transcriptional and translational silencing due to compacted DNA and absence of organelles (Ren et al., 2017). Without new RNA produced in the mature sperm before and after freezing, only differential degradation amount of RNA that was already synthesized and stored prior to transcriptional arrest was taken into account (Lalancette et al., 2008). For instance, the m^6^A modification can regulate the stabilization, degradation, and translation of mRNA (Wang et al., 2015; Edupuganti et al., 2017; Qin et al., 2021). Thus, significant changes in methylation modifications on sperm transcripts caused by freezing could result in up- and downregulation of mRNA at the transcriptome level due to possible differences in mRNA degradation rates between the fresh and frozen semen. As expected, numerous studies have also identified several up- and downregulated transcripts in the frozen semen (Dai et al., 2019; Wang et al., 2022), and a previous study has indicated that the number of up- and downregulated genes in bull frozen semen compared with the fresh semen was 241 and 662, respectively (Ebenezer Samuel King et al., 2022). In addition, numerous past studies have also explored potential changes in mRNA and protein levels in the frozen semen of various species, including boar (Chen et al., 2014; Dai et al., 2019; Fraser et al., 2020), human (Wang et al., 2022), and sheep (Jia et al., 2022) by transcriptomics or proteomics. For example, in the transcriptome analysis of the fresh and frozen boar semen, differentially expressed mRNA and miRNA were found to be mainly related to environmental stress, apoptosis, and metabolism (Dai et al., 2019). Comparative transcriptomics analysis has revealed that compared with the fresh sperm, DEGs of human frozen and vitrified sperm were associated with different immune and infectious diseases (Wang et al., 2022). These findings further corroborate with the results of our study that the enriched pathways could be mainly categorized into metabolism, immune response, disease, and signal transduction. Comparative proteomics analysis of the fresh and frozen boar semen has revealed that most of the DEPs primarily participated in the sperm premature capacitation, adhesions, energy supply, and sperm–oocyte binding and fusion (Chen et al., 2014). Isobaric tags for the relative and absolute quantification combined with parallel reaction monitoring (PRM) proteomics analysis of the fresh and frozen sheep semen indicated that DEPs were enriched in metabolic activities, disease, and oxidative phosphorylation pathways (Jia et al., 2022). In this study, DEPs were mainly involved in the heterogeneous metabolic pathways. The variability between the studies could be attributed to the possible differences in experimental species, freezing procedures, and detection methods.

**FIGURE 11:**
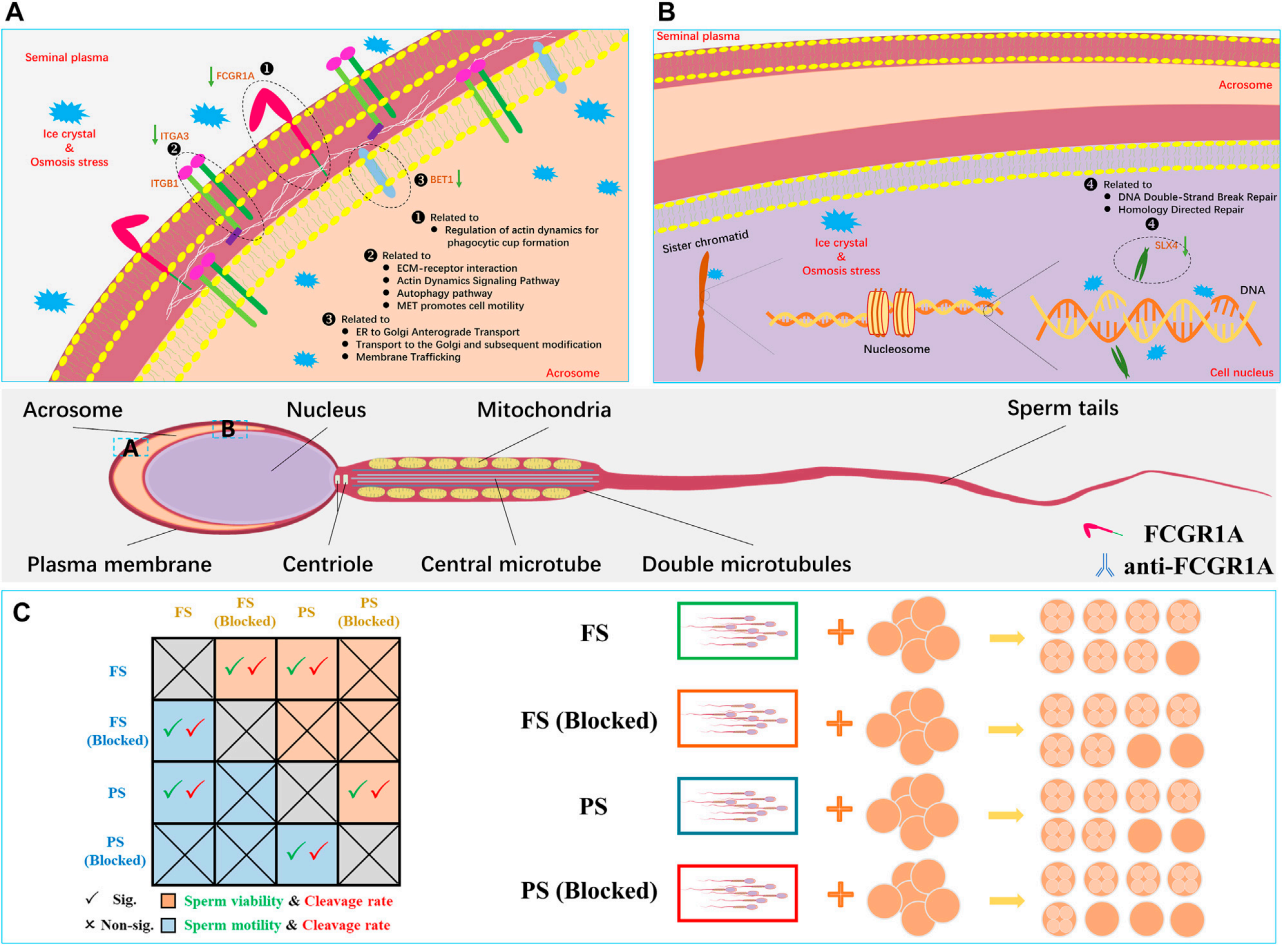
Representative sperm mRNA and protein response mechanisms in the frozen semen of sheep. **(A)** Located in the sperm acrosomal and cytoplasm membrane, FCGR1A, ITGA3, and BET1 proteins are downregulated related to the pathway and function. **(B)** Located in the sperm nucleus, SLX4 is downregulated related to the pathway and function. **(C)** Schematic diagram showing the differences in sperm motility, viability, cleavage rate blocked, and unblocked sperm.

For in-depth analysis, integrated Co-KEGG enrichment analysis and Pearson’s correlation analysis were conducted, and the results revealed that *FCGR1A*, *ITGA3*, *SLX4*, and *BET1* gene clusters were correlated with 11 distinct DEPs (Figures 11A,B). Our findings indicated that *FCGR1A* was more than a member of DEG/DEP as it could also associate with the highest number of proteins in Pearson’s correlation analysis. Interestingly, a prior report has indicated that *FCGR1A* was involved in the modulation of immune response and mainly expressed on the cell membrane of leukocytes (Barb, 2021). Additionally, in neutrophilic granulocytes, *FCGR1A* can act as a receptor for signal transduction and induce dynamic changes in kinase expression, which can finally result in cell morphology modification to enhance phagocytosis (Futosi et al., 2013). Furthermore, in the monocytes, the binding of *FCGR1A* to the ligands can lead to phagocytosis and promote the release of various inflammatory factors and reactive oxygen species (Swisher and Feldman, 2015). Moreover, a previous study has reported that *FCGR1A* was located on the sperm plasma membrane (Hellstrom et al., 1988). However, the function of *FCGR1A* in sperm fertilization remains undetermined until now. In the present study, ITGA3 was also associated with DEPs. ITGA3, a member of a huge integrin family, can mainly combine with ITGB1 to form a complete integrin and play a vital role in sperm adhesion and sperm–oocyte fusion (Merc et al., 2021). It has been reported that the integrin could act as an essential transmembrane protein and play an important role in the signal transduction process by interacting with intracellular kinases or acting indirectly with actin (Green and Brown, 2019). Therefore, we hypothesized that the downregulated ITGA3 by freezing might inhibit the binding of the frozen sperm to the oocytes. Interestingly, SLX4 was identified to be correlated with DEPs. Another previous study has shown that the SLX1–SLX4 complex plays a vital role in maintaining the genome stability, including preventing the collapse of the replication forks and cleaving Holliday junctions (Fricke and Brill, 2003; Garner et al., 2013). Xu et al. (2021) discovered that SLX1 could process 5′-flap DNA efficiently and rapidly primarily by cooperating with SLX4 in the presence of the SAP domain. In addition, it has been reported that increased sperm DNA fragmentation was found in the frozen sheep sperm (Palazzese et al., 2018), and the phenomenon might be explained by the reduced *SLX4* transcript discovered in the present study since SLX4 could contribute to the DNA instability. In addition, BET1 was identified as another vital gene associated with DEPs. For example, a prior study has indicated that BET1 could be involved in endoplasmic reticulum-secreted vesicle recognition, fusion, and molecule transport to the Golgi apparatus (Zhang et al., 1997; Malsam and Söllner, 2011). It is well known that the acrosome is developed from the Golgi apparatus and contains diverse digestive enzymes required for sperm penetration (Khawar et al., 2019). As a membrane protein, BET1 probably can integrate into the Golgi apparatus, which may eventually evolve into the acrosome membrane, and the reduced BET1 content might impair sperm fertilization capability.

Moreover, we found that there were 11 proteins associated with four genes under Pearson’s correlation analysis. Interestingly, most of these 11 proteins were associated with sperm motility and fertilization capacity. In the reproductive system, MME has been associated with sperm formation and development and can regulate follicle maturation and ovulation (Nalivaeva et al., 2020). However, its specific mechanism remains unclear. It has been reported that the addition of the inhibitor (thiorphan) to inhibit CD10 (also called MME) can significantly enhance human sperm motility (Subirán et al., 2010). However, the reduction in sperm motility caused by freezing was observed to be accompanied by the downregulation of MME, which requires further research. L-PGDS (also called PTGDS) can mainly localize in the head of the epididymis and in the apical ridge of the acrosome (Gerena er al., 2000). Analysis by ELISA and flow cytometry indicated that L-PGDS can improve sperm motility (Chen et al., 2007). The bovine sperm or oocytes treated with the L-PGDS antibody could improve the sperm–oocyte binding, thereby reducing the fertilization ability (Gonçalves et al., 2008). In addition, compared with the semen with the highest freezability, L-PGDS with the lowest freezability showed a decreasing trend in boar frozen semen (Valencia et al., 2017). In this study, downregulated PTGDS could potentially induce polyspermism, thereby resulting in decreased oocyte quality and fertilization failure of frozen sperm. Male mice with knocked-out HEXB can exhibit a lower fertilization rate at approximately 109.2 days of age compared to wild-type mice (Juneja, 2002). Thus, we believed that the downregulated HEXB could be the probable reason for the low fertilization rate observed in the frozen semen. A high-throughput phenotypic screening platform has revealed that sperm motility was elevated when PDE10A was inhibited (Gruber, et al., 2022). For instance, in the brain, PDE10A has been reported to be involved in the regulation of cAMP and cGMP synthesis and is commonly used as a therapeutic inhibitor for psychiatric disorder treatment (Wilson and Brandon, 2015). Since cAMP signaling pathway and calcium ions can play crucial roles in sperm capacitation and motility acquisition (Balbach et al., 2018), it was hypothesized that the downregulation of PDE10A in the frozen group could be responsible for reduced mobility in the frozen sperm. During fertilization, the GALT protein present on the sperm membrane can adhere to zona pellucida 3 (ZP3) on the oocyte membrane (Shur et al., 2006). Thus, the downregulated GALT in the frozen sperm might reduce the sperm–oocyte adhesion in the pre-acrosome reaction. It was demonstrated that the expression of HSPA1L was significantly reduced in the low-motility sperm, and the antibody neutralization experiment indicated that blocking HSPA1L could significantly reduce the sperm motility (Liu et al., 2022). Moreover, a previous study has indicated that stress can lead to the activation of HSPA1L (Murphy, 2013). Therefore, we hypothesized that the low HSPA1L levels in the frozen sperm could also attribute to the reduced sperm motility in our study. It has been reported that CTSD could be transported from the epididymis to the surface of sperm during sperm maturation (Asuvapongpatana et al., 2013). A previous study has also indicated that CTSD in the human sperm could be activated during capacitation (Saewu et al., 2012). However, in the present study, reduced CTSD abundance was found in the frozen sperm, which could possibly disturb sperm fertilization through impaired sperm capacitation. Notably, *FCGR1A* was the only gene identified upon conducting the three integrated multi-omics analyses, and it was found to be downregulated in both RNA-seq and proteome profiles. FCGR1A, also known as CD64 (FCGR1A), is a member of the differentiation (CD) family. CD proteins are mainly localized on the surface of immune cells and play vital roles in signal recognition during immune response. Notably, numerous studies have revealed that CD9 protein belonging to this family is associated with sperm–oocyte binding and fusion (Siu et al., 2021). The results of a binding assay using radio-iodinated immunoglobulin showed that both human and mouse sperm possessed Fc receptors on the surface, which could effectively bind to the Fc *γ* region (Sethi and Brandis, 1980). Additionally, another study has indicated that human seminal plasma containing Fc *γ* receptor proteins could significantly protect sperm from immune damage in the female reproductive tract (Chiu and Chamley, 2003). In this report, we have demonstrated that FCGR1A abundance is directly related to sperm motility and viability. Sperm-blocking experiments further revealed that the loss of FCGR1A could effectively reduce sperm viability and motility and also result in the decreased cleavage rate (Figure 11C). In addition, evidence has indicated that FCGR1A could affect diverse biological events, including cytoskeletal changes, ROS production, and cellular survival, through modulating SYK, SYK partners, and other signaling intermediates (Mócsai et al., 2010; van der Poel et al., 2011). Interestingly, a previous finding has identified that FCGR (including FCGR1A) recruitment is required for SYK-mediated cytoskeleton reorganization (Jaumouillé et al., 2014). It was demonstrated that the sperm motility is primarily dependent on sperm tail motility, which is maintained by the normal cytoskeletal axonemal structure and adequate ATP supply in a constant and stable manner (Lehti and Sironen, 2017). Thus, the low-content FCGR1A could possibly cause insufficient recruitment and further disturb the cytoskeleton reorganization, and hence we hypothesized that the downregulated FCGR1A expression in the frozen sperm could lead to reduced motility by interrupting the cytoskeletal organization. Moreover, it has been discovered that Fc *γ* receptors also existed on the oocyte membrane surface, and the binding of the ligand to the receptor is required for the correct signal transduction during sperm recognition in the fertilization process (Bronson, 1998). Moreover, another recent study has highlighted that Fc receptor-like 3 located on the oocyte membrane could also act as a binding receptor similar to the JUNO and IZUMO1 proteins (Vondrakova et al., 2022). Overall, we speculate that FCGR1A might also play a role in sperm–egg recognition, which needs to be further validated in future studies.

## 5 Conclusion

In summary, our results have demonstrated that integrated multi-omics could be effectively used as a potent tool in identifying various candidate genes responsible for sperm cryoinjuries. Moreover, we have discovered that downregulated FCGR1A could result in reduced motility and viability, which can further attribute to the compromised fertility capacity in frozen sheep sperm.

## Data Availability

This raw data of this article will be available by the iProX (Project ID: PXD044096) and National Genomics Data Center with identifier (Project ID: PRJCA018587).
